# Supplementary material for: Transcripts switched off at the stop of phloem unloading highlight the energy efficiency of sugar import in the ripening V. vinifera fruit
Source: Hortic Res. 2021 Sep 1;8:193. doi: 10.1038/s41438-021-00628-6 (PMC8408237; doi:10.1038/s41438-021-00628-6)
Supplement: Supplementary file 2 — Table S1. [file 41438_2021_628_MOESM2_ESM.pdf]

Table S1: Evolution of sugars, acid and cations. (a) Amounts per fruit (concentration x volume), (b) concentrations and (c) net H+/sucrose exchange escaping V-ATPase at the tonoplast.

A	Stage	Day	Volume Increment	Glucose (mM)	Fructose (mM)	Tartrate (mEq)	Malate (mEq)	K+ (mEq)	G+F	G/F	M/T
			Average ± Standard error								
**SYRAH**											
	**V**	0	1.00 ± 0.00	73.72 ± 2.46	34.21 ± 1.77	87.11 ± 2.15	186.74 ± 5.07	23.61 ± 0.52	107.94 ± 4.12	1.29 ± 0.03	1.56 ± 0.09
	**G**	13	1.53 ± 0.02	414.26 ± 9.86	353.60 ± 9.41	95.02 ± 3.16	104.85 ± 5.05	25.78 ± 0.54	767.86 ± 19.25	1.06 ± 0.01	1.00 ± 0.05
	**P**	23	1.70 ± 0.02	646.09 ± 10.87	558.77 ± 9.35	92.59 ± 2.13	65.86 ± 2.55	36.39 ± 0.85	1204.87 ± 20.19	1.16 ± 0.01	0.72 ± 0.04
	**S**	36	1.58 ± 0.03	604.67 ± 12.67	519.40 ± 10.09	86.11 ± 2.12	38.62 ± 1.53	35.38 ± 1.04	1124.06 ± 22.73	1.08 ± 0.02	0.42 ± 0.02
**MV032**											
	**V**	0	1.00 ± 0.00	39.27 ± 2.48	19.42 ± 1.68	74.52 ± 1.07	210.91 ± 8.55	17.73 ± 0.48	58.69 ± 4.01	1.13 ± 0.06	1.53 ± 0.08
	**G**	15	1.55 ± 0.01	267.25 ± 6.95	266.18 ± 7.73	67.26 ± 1.26	98.22 ± 7.24	36.54 ± 0.71	533.43 ± 14.62	0.84 ± 0.01	1.22 ± 0.09
	**P**	27	1.86 ± 0.03	391.67 ± 18.94	402.47 ± 19.09	74.99 ± 1.37	68.39 ± 4.16	48.32 ± 1.85	794.13 ± 37.99	0.97 ± 0.02	0.91 ± 0.05
	**S**	40	1.76 ± 0.03	396.59 ± 8.50	418 ± 9.56	75.37 ± 1.44	47.65 ± 1.83	50.35 ± 1.45	814.59 ± 18.00	0.90 ± 0.02	0.60 ± 0.03
**MV102**											
	**V**	0	1.00 ± 0.00	43.55 ± 4.51	25.09 ± 3.88	49.44 ± 0.73	237.51 ± 3.96	21.66 ± 0.47	68.65 ± 8.37	1.12 ± 0.07	2.84 ± 0.06
	**G**	16	1.50 ± 0.01	282.46 ± 6.76	267.47 ± 6.97	48.18 ± 0.93	143.93 ± 5.32	47.08 ± 0.62	549.93 ± 13.72	0.94 ± 0.01	2.66 ± 0.12
	**P**	22	1.69 ± 0.00	372.34 ± 7.40	360.81 ± 8.57	59.58 ± 1.53	115.52 ± 7.04	57.37 ± 2.43	733.15 ± 15.93	1.03 ± 0.00	1.94 ± 0.09
	**S**	36	1.52 ± 0.03	386.14 ± 10.23	382.84 ± 11.48	59.03 ± 1.61	92.46 ± 6.19	60.04 ± 1.18	768.98 ± 21.57	0.90 ± 0.02	1.41 ± 0.10

B

	Stage	Day	Volume Increment	Glucose (mM)	Fructose (mM)	Tartrate (mEq)	Malate (mEq)	K+ (mEq)	G+F	G/F	M/T
			**Average ± Standard error**								
**SYRAH**											
	**V**	0	1.00 ± 0.00	125.02 ± 4.18	58.02 ± 3.01	147.73 ± 3.65	316.69 ± 8.59	40.03 ± 0.88	183.04 ± 6.99	2.19 ± 0.06	2.64 ± 0.15
	**G**	13	1.53 ± 0.02	459.11 ± 10.30	391.83 ± 9.81	105.19 ± 3.18	116.06 ± 5.24	28.60 ± 0.66	850.93 ± 20.08	1.17 ± 0.00	1.11 ± 0.05
	**P**	23	1.70 ± 0.02	646.09 ± 7.90	558.78 ± 6.84	92.64 ± 1.97	65.79 ± 2.26	36.37 ± 0.69	1204.87 ± 14.69	1.16 ± 0.00	0.72 ± 0.04
	**S**	36	1.58 ± 0.03	650.97 ± 10.32	559.26 ± 8.21	92.70 ± 1.90	41.48 ± 1.33	38.03 ± 0.85	1210.23 ± 18.48	1.16 ± 0.00	0.45 ± 0.02
**MV032**											
	**V**	0	1.00 ± 0.00	73.02 ± 4.61	36.11 ± 3.12	138.57 ± 2.00	392.19 ± 15.91	32.97 ± 0.89	109.13 ± 7.46	2.09 ± 0.11	2.85 ± 0.14
	**G**	15	1.55 ± 0.01	321.42 ± 8.14	320.23 ± 9.34	80.86 ± 1.38	117.80 ± 8.10	43.95 ± 0.86	641.65 ± 17.42	1.01 ± 0.01	1.46 ± 0.10
	**P**	27	1.86 ± 0.03	390.06 ± 14.06	400.80 ± 13.89	75.18 ± 1.75	68.82 ± 4.63	48.20 ± 1.27	790.86 ± 27.89	0.97 ± 0.00	0.91 ± 0.05
	**S**	40	1.76 ± 0.03	419.06 ± 6.86	441.67 ± 7.95	79.74 ± 1.68	50.27 ± 1.58	53.18 ± 1.26	860.73 ± 14.73	0.95 ± 0.00	0.63 ± 0.02
**MV102**											
	**V**	0	1.00 ± 0.00	73.79 ± 7.65	42.51 ± 6.58	83.76 ± 1.23	402.38 ± 6.72	36.70 ± 0.80	116.30 ± 14.18	1.89 ± 0.12	4.81 ± 0.10
	**G**	16	1.50 ± 0.01	319.20 ± 7.47	302.24 ± 7.67	54.42 ± 0.89	162.59 ± 5.83	53.22 ± 0.72	621.44 ± 15.13	1.06 ± 0.00	3.00 ± 0.13
	**P**	22	1.69 ± 0.00	372.28 ± 6.48	360.73 ± 7.63	59.60 ± 1.65	115.53 ± 7.07	57.35 ± 2.30	733.01 ± 14.06	1.03 ± 0.01	1.93 ± 0.09
	**S**	36	1.52 ± 0.03	432.23 ± 9.91	428.04 ± 9.74	66.05 ± 1.42	104.86 ± 9.14	67.28 ± 1.31	860.28 ± 19.39	1.01 ± 0.01	1.60 ± 0.15

C		net influx (mMol or mEq)			net exchange		
		**sucrose**	**malate**	**K+**	**H+/ sucrose**	**K+/ sucrose**	**(H+-K+)/ sucrose**
**SYRAH**							
	**from V to G**	330	-82	2	-0.25	0.01	-0.25
	**from G to P**	219	-39	11	-0.18	0.05	-0.23
	**from V to P**	548	-121	13	-0.22	0.02	-0.24
**MV032**	**from V to G**	237	-113	19	-0.47	0.08	-0.55
	**from G to P**	130	-30	12	-0.23	0.09	-0.32
	**from V to P**	368	-143	31	-0.39	0.08	-0.47
**MV102**	**from V to G**	241	-94	25	-0.39	0.11	-0.49
	**from G to P**	92	-28	10	-0.31	0.11	-0.42
	**from V to P**	332	-122	36	-0.37	0.11	-0.47
