# Supplementary material for: Transcripts switched off at the stop of phloem unloading highlight the energy efficiency of sugar import in the ripening V. vinifera fruit
Source: Hortic Res. 2021 Sep 1;8:193. doi: 10.1038/s41438-021-00628-6 (PMC8408237; doi:10.1038/s41438-021-00628-6)
Supplement: Supplementary file 3 — Table S5. [file 41438_2021_628_MOESM3_ESM.pdf]

Table S5. Depletion of cytosolic sugar at equilibrium state of vacuolar transports, in fruit parenchymal cells from the onset to the arrest of sugar loading.

Change in Gibbs energy for sucrose hydrolysis: $S+H_2O \leftrightarrow G+F$ $\Delta G_0 = -29.6$ kJ.mol^{-1}

where G, F and S stand for glucose, fructose and sucrose, respectively.

(Putnam and Boerio-Goates, 1993, *J. Chem. Thermodynamics*, 25, 605-613)

Since $\ln(K_{eq}) = -\Delta G_0/RT$, at 25°C it comes : $([G] \times [F]) / [(S] \times [H_2O]) = 1.5\ 10^5$

A berry at the stop of phloem includes within 1 mol.l^{-1} G+F and ca 50 mol.l^{-1} H_2O at pH = 3.5, hence S = 33 nmol.l^{-1} in the vacuole, when sucrose hydrolysis by GIN1 reaches equilibrium state.

Hypothesis 1: Sucrose/H^+ exchange at the tonoplast: $S_{cyt} + H^+_{vac} \leftrightarrow S_{vac} + H^+_{cyt}$

assuming $pH_{cyt} = 7.5$ and $pH_{vac} = 3.5$ (ripe stage): $S_{cyt} / S_{vac} = H^+_{cyt} / H^+_{vac} = 10^{(pHvac-pHcyt)} = 10^{-4}$

$pH_{vac} = 2.7$ (green stage) : $S_{cyt} / S_{vac} = H^+_{cyt} / H^+_{vac} = 10^{(pHvac-pHcyt)} = 10^{-4.8}$

Conclusion: At ripe stage, only $3\ 10^{-12}$ mol.l^{-1} sucrose remains in the cytoplasm, when TST and vacuolar invertase reach thermodynamical equilibrium. Cytosolic sucrose should even be 100 fold less concentrated at the onset of ripening, when $pH_{vac} = 2.7$, $G_{vac} = 100$ mM and $F_{vac} < 50$ mM. Such a virtual elimination of sucrose from the cytoplasm may explain the huge activities of sucrose related enzymes like SuSy and SPS.

Hypothesis 2: Hexose/H^+ exchange at the tonoplast: $G_{cyt} + H^+_{vac} \leftrightarrow G_{vac} + H^+_{cyt}$

$F_{cyt} + H^+_{vac} \leftrightarrow F_{vac} + H^+_{cyt}$

at equilibrium state : $G_{cyt}/G_{vac} = F_{cyt}/F_{vac} = H^+_{cyt}/H^+_{vac} = 10^{(pHvac-pHcyt)}$

ripe stage , $pH_{vac} < 3.7$, $G_{vac} = F_{vac} = 0.5$ mol.l^{-1} , thus $G_{cyt} = F_{cyt} < 50$ µmol.l^{-1}

onset of ripening, $pH_{vac} = 2.7$, $F_{vac} < G_{vac} < 0.12$ mol.l^{-1} , thus $F_{cyt} < G_{cyt} < 1.2$ µmol.l^{-1}

General conclusion: At equilibrium state, the most conservative hypothesis (no positive inside vacuolar membrane potential, hexose/sugar antiport quite inconsistent with the activity of vacuolar invertase, and the presence of sucrose in *Vitis* species lacking this enzyme) show that there is no need to energize sugar transport using H^+ sugar symporters at the plasma membrane, when apoplasmic sugar exceed 50 µM.
