# Supplementary material for: Transcripts switched off at the stop of phloem unloading highlight the energy efficiency of sugar import in the ripening V. vinifera fruit
Source: Hortic Res. 2021 Sep 1;8:193. doi: 10.1038/s41438-021-00628-6 (PMC8408237; doi:10.1038/s41438-021-00628-6)
Supplement: Supplementary file 4 — Table S6. [file 41438_2021_628_MOESM4_ESM.pdf]

Table S6: RNA-Sequencing data metrics

Variety	Stage	Replicate	Reads sequences	Reads after Trimming	Reads aligned 12X2	Reads mapped
Syrah	G	1	30317743	24196863	22379920	21746226
Syrah	G	2	38767502	31617671	29329953	28201855
Syrah	G	3	36902092	30975326	28557053	27390403
Syrah	P	1	48825050	37836874	32032842	29884800
Syrah	P	2	43928122	39020281	35816007	34053210
Syrah	P	3	33035742	28346568	25576427	24515999
Syrah	S	1	29379364	26673004	24287791	23609172
Syrah	S	2	27932074	25346878	23635948	23142294
Syrah	S	3	24753333	21488277	18424304	17567192
MV032	G	1	37778952	34558638	31498665	29601322
MV032	G	2	36531987	34113094	31123253	29165529
MV032	G	3	28840223	26523513	22105592	20228613
MV032	P	1	20907445	18987562	16786570	15430250
MV032	P	2	38606492	35458254	33141315	31455223
MV032	P	3	28356906	25148666	24036548	22903362
MV032	S	1	40005534	36967813	33486063	31591865
MV032	S	2	34997496	32024644	28907918	27281216
MV032	S	3	34003160	31401494	24977157	22160556
MV102	G	1	27029143	24339855	22912916	21545283
MV102	G	2	34021450	31084770	29231874	27353929
MV102	G	3	23425399	21734491	20489657	19040850
MV102	P	1	36376192	32441228	30373853	28489111
MV102	P	2	21335382	19352056	18071993	16855681
MV102	P	3	18994808	16793468	15803340	14814356
MV102	S	1	23338681	21476137	19943800	18793897
MV102	S	2	23647110	21516269	20352925	19244831
MV102	S	3	31880416	28854330	26902030	25386387
